# Hepatic Gene Expression Profile in Mice Perorally Infected with *Echinococcus multilocularis* Eggs

**DOI:** 10.1371/journal.pone.0009779

**Published:** 2010-04-01

**Authors:** Bruno Gottstein, Matthias Wittwer, Marc Schild, Michael Merli, Stephen L. Leib, Norbert Müller, Joachim Müller, Rolf Jaggi

**Affiliations:** 1 Vetsuisse Faculty, Institute of Parasitology, University of Bern, Bern, Switzerland; 2 Faculty of Medicine, Institute for Infectious Diseases, University of Bern, Bern, Switzerland; 3 Department of Parasitology, University of Hohenheim, Stuttgart, Germany; 4 Faculty of Medicine, Department of Clinical Research, University of Bern, Bern, Switzerland; Charité-Universitätsmedizin Berlin, Germany

## Abstract

**Background:**

Alveolar echinococcosis (AE) is a severe chronic hepatic parasitic disease currently emerging in central and eastern Europe. Untreated AE presents a high mortality (>90%) due to a severe hepatic destruction as a result of parasitic metacestode proliferation which behaves like a malignant tumor. Despite this severe course and outcome of disease, the genetic program that regulates the host response leading to organ damage as a consequence of hepatic alveolar echinococcosis is largely unknown.

**Methodology/Principal Findings:**

We used a mouse model of AE to assess gene expression profiles in the liver after establishment of a chronic disease status as a result of a primary peroral infection with eggs of the fox tapeworm *Echinococcus multilocularis*. Among 38 genes differentially regulated (false discovery rate adjusted p≤0.05), 35 genes were assigned to the functional gene ontology group <immune response>, while 3 associated with the functional group <intermediary metabolism>. Upregulated genes associated with <immune response> could be clustered into functional subgroups including <macrophages>, <APCs>, <lymphocytes, chemokines and regulation>, <B-cells> and <eosinophils>. Two downregulated genes related to <lymphocytes, chemokines and regulation> and <intermediary metabolism>, respectively. The <immune response> genes either associated with an <immunosupression> or an <immunostimulation> pathway. From the overexpressed genes, 18 genes were subsequently processed with a Custom Array microfluidic card system in order to assess respective expression status at the mRNA level relative to 5 reference genes (Gapdh, Est1, Rlp3, Mdh-1, Rpl37) selected upon a constitutive and stable expression level. The results generated by the two independent tools used for the assessment of gene expression, i.e., microarray and microfluidic card system, exhibited a high level of congruency (Spearman correlation rho = 0.81, p = 7.87e-5) and thus validated the applied methods.

**Conclusions/Significance:**

Based on this set of biomarkers, new diagnostic targets have been made available to predict disease status and progression. These biomarkers may also offer new targets for immuno-therapeutic intervention.

## Introduction

Alveolar echinococcosis (AE) is a result of a hepatic infection with the larval (metacestode) stage of the fox tapeworm *Echinococcus multilocularis*. AE as a disease is associated with high mortality (>90%) if remaining untreated [1]. AE patients are affected by hepatic sequelae that are due to a wide spectrum of liver injury leading predominantly to cholestatic jaundice (about a third of cases) and/or unspecific epigastric pain (about a third of cases), together with various symptoms such as fatigue, weight loss and hepatomegaly [2].

As a result of peroral infection via eggs of *E. multilocularis*, the parasitic metacestode (larval stage) subsequently grows as a tumor-like tissue in the liver of its intermediate host, include predominantly small rodents, but accidentally also humans. Thus, the laboratory mouse is an excellent model to study the host-parasite interplay. While most studies so far have been carried out upon so-called secondary infections (intraperitoneal inoculation of fully developed metacestode tissue), the respective difficulty lies in the fact that this infection model does not include primary hepatic events that are crucial to understand the natural host-parasite interplay. The real approach to determining the biological events in vivo is to carry out peroral inoculation of infectious *E. multilocularis* eggs, experiments that can only be performed in appropriate biosafety level 3 laboratory units. Such experimental infection is referred to as primary infection, resulting in an intrahepatic tumor-like growth of the metacestode that overcomes the immune system and subsequently establishes a chronic phase of infection, which persists approximately between 4–6 months p.i.. Through effects on cells of both the innate and adaptive arms of the immune response, the parasite can orchestrate a range of outcomes that are beneficial not only for metacestode establishment, but also in terms of facilitating its proliferation and maturation. In addition, the complex host-parasite interaction leads to only limited pathology. Likewise, a higher survival potential for both host and parasite is achieved.

Despite the severity of AE in humans, the genetic program that regulates the mechanisms leading to liver damage as a consequence of AE is largely unknown. High-throughput methods, e.g. DNA microarrays, can provide a comprehensive picture of the genes underlying the host responses to AE. This knowledge is a prerequisite for understanding the pathogenesis of liver damage and can drive the development of new prognostic and/or therapeutic modalities for AE. The aim of this study was to identify genes and sets of genes implicated in the immuno-pathophysiological mechanisms leading to the induction of a host response to infection, but subsequently also to hepatic damage observed in experimental AE. We used an appropriate mouse model of primary AE infection and DNA microarray technology to assess gene expression profiles in the periparasitic liver tissue known to be preferentially affected, in mock-infected controls and during the phase of early chronic AE following peroral infection of the mice with infectious *E. multilocularis* eggs (thus exactly mimicking the natural way of infection). Significantly overexpressed genes on microarrays were re-investigated and validated by real-time RT-PCR using microfluidic cards.

## Results

### Animal model

Eight to 10-week-old female BALB/c mice were purchased from Charles River GmbH, Germany. For all experiments, animals were matched for age and weight. All mice were housed and handled under standard aseptic animal laboratory conditions according to the rules of the Swiss regulations for animal experimentation. Maintenance of perorally *E. multilocularis* egg infected animals (see below) was carried out in a B3 safety containment, these experiments required governmental safety approval (Swiss Federal concession no. A990006/3A). Primary infections of mice were all based upon the use of a single batch of *E. multilocularis* eggs, obtained and purified as previously described [3]. The viability and infectivity rate of this batch of eggs had been predetermined by appropriate explorative titration-infection trials in mice [4]. For the present batch and experiments, primary infection parameters were 2,000 eggs per mouse to be administered perorally, yielding a medium number of 26 primary lesions per liver (range 12–35). Technically, intragastric *E. multilocularis* egg inoculation was performed as described elsewhere [5]. 31 days after infection, all infected animals (n = 8) had alveolar echinococcosis of the liver as evidenced by the presence of between 5 to 22 hepatic liver lesions, all exhibiting the same morphology including a central parasitic vesicle of approximately 1–2 mm of diameter, and surrounded by a white periparasitic inflammatory corona of about 0.5 mm in diameter. Mock-infected control animals presented neither macroscopically nor microscopically visible lesions in the liver.

### Microarrays

Changes of the mouse hepatic gene expression in response to primary hepatic *E. multilocularis* infection were examined during the initial phase of chronic infection stage (i.e. after 1 month). The parasitized animals shared a total of 38 genes that significantly changed after 1 mon of infection (fdr adjusted P-value of <0.05) (Tab. 1). Of those genes, 36 appeared upregulated in reference to non-infected controls, and 2 yielded down-regulation.

**Table 1:** Probe-sets representing 38 genes, their expression levels in the liver, organized according to their functional groups.

#	AFFY ID	REFSEQ ID	GENE	NCBI Gene ID	DESCR	*ratio*	expr_AE-LIVER	expr_LIVER mock-infected
**OVEREXPESSED/UPREGULATED**								
*Immune response/defense* [functional group]: Macrophages (MØ)								
01	1425394_at	XP_001471630	BC023105	667597	cDNA sequence aBC023105 p47-like GTPase	*10*	17	169
02	**1449009_at**	NM_011579	Tgtp	21822	T-cell specific GTPase	*6.6*	302	2001
03	1429184_at	NM_001039160	Gvin1	74558	GTPase, very large interferon inducible 1	*3.3*	293	954
04	1424923_at	NM_009251	Serpina3g	20715	serine (or cysteine) peptidase inhibitor, member 3G	*6.4*	84	534
05	1420549_at	NM_010259	Gbp1	14468	guanylate binding protein 1	*5.0*	24	118
06	1435906_x_at	NM_010260	Gbp2	14469	guanylate binding protein 2	*3.0*	93	277
07	1418776_at	NM_029509	5830443L24Rik	76074	RIKEN cDNA 5830443L24 gene, GBP-like	*3.4*	91	310
08	**1449498_at**	NM_010766	Marco	17167	macrophage receptor with collagenous structure	*4.7*	51	236
09	1447927_at	NM_194336	Mpa2l	100702	macrophage activation 2 like	*3.6*	342	1223
10	**1440481_at**	NM_009283	Stat1	20846	signal transducer and activator of transcription 1	*3.4*	36	121
11	1436808_x_at	NM_008566	Mcm5	17218	minichromosome maintenance deficient 5,	*2.9*	31	91
12	**1420699_at**	NM_020008	Clec7a	56644	C-type lectin domain family 7, member a	*2.8*	70	193
13	**1416295_a_at**	NM_013563	Il2rg	16186	interleukin 2 receptor, gamma chain	*2.8*	67	189
14	**1426808_at**	NM_010705	Lgals3	16854	lectin, galactose binding, soluble 3, galektin	*2.6*	118	300
15	1448748_at	NM_019549	Plek	56193	pleckstrin	*2.4*	29	70
*Immune response/defense* [functional group]: APC								
16	1450648_s_at	NM_207105	H2-Ab1	14961	histocompatibility 2, class II antigen A, beta 1	*5.6*	230	1287
17	**1417025_at**	NM_010382	H2-Eb1	14969	histocompatibility 2, class II antigen E beta	*5.3*	151	796
18	1435290_x_at	NM_010378	H2-Aa	14960	histocompatibility 2, class II antigen A, alpha	*4.4*	457	1999
19	**1422527_at**	NM_010386	H2-Dma	14998	histocompatibility 2, class II, locus Dma	*2.9*	31	90
20	**1418638_at**	NM_010387	H2-DMb1	14999	histocompatibility 2, class II, locus Mb1	*2.9*	15	43
21	**1425519_a_at**	NM_001042605	Cd74	16149	CD74 antigen (invariant polypeptide of major histo compatibility complex, class II antigen-associated)	*3.8*	602	2268
*Immune response/defense* [functional group]: Lymphocytes, chemokines and regulation								
22	**1418652_at**	NM_008599	Cxcl9	17329	chemokine (C-X-C motif) ligand 9	*5.2*	254	1328
23	**1418126_at**	NM_207105	Ccl5	20304	chemokine (C-C motif) ligand 5	*4.5*	24	111
24	**1418930_at**	NM_021274	Cxcl10	15945	chemokine (C-X-C motif) ligand 10	*4.1*	81	327
25	**1423467_at**	NM_021718	Ms4a4b	60361	membrane-spanning 4-domains, subfamily A, CD20	*3.0*	25	74
26	1424638_at	NM_001111099	Cdkn1a	12575	cyclin-dependent kinase inhibitor 1A (P21)	*2.8*	22	63
27	1452349_x_at	NM_172648	Ifi205	226695	interferon activated gene 205	*2.6*	188	492
28	**1422903_at**	NM_010745	Ly86	17084	lymphocyte antigen 86	*2.4*	211	506
29	**1421855_at**	NM_008013	Fgl2	14190	fibrinogen-like protein 2	*2.4*	37	88
30	**1448162_at**	NM_011693	Vcam1	22329	vascular cell adhesion molecule 1	*2.2*	118	259
31	1447830_s_at	NM_009061	Rgs2	19735	regulator of G-protein signaling 2	*2.3*	29	67
*Immune response/defense* [functional group]: B-cells								
32	1427455_x_at	XM_001476664	Igk-V1	16081	immunoglobulin kappa chain variable 1 (V1)	*4.7*	90	420
33	1452417_x_a	XM_001476664	Igk-C	16071	immunoglobulin kappa chain, constant region	*4.4*	130	574
*Immune response/defense* [functional group]: Eosinophils								
34	1449846_at	NM_007895	Ear2	13587	eosinophil-associated, ribonuclease A family, member 2	*2.6*	41	104
*Intermediary metabolism* [functional group]: hepatocytes								
35	**1431056_a_at**	NM_008509	Lpl	16956	lipoprotein lipase	*2.6*	358	975
36	1442798_x_at	NM_001033245	Hk3	212032	hexokinase 3	*2.4*	37	90
**DOWNREGULATED**								
*Immune response/defense* [functional group]: Lymphocytes, chemokines and regulation								
37	1451069_at	NM_145478	Pim3	223775	proviral integration site 3	*0.4*	298	118
*Intermediary metabolism* [functional group]: hepatocytes								
38	1451588_at	NM_026947	1810022C23Rik	69123	RIKEN cDNA 1810022C23 gene, Crotonase-like	*0.4*	278	100

In bold (AFFY ID nos.) are genes that subsequently were subjected to retesting by a microfluidic card system. Genes were selected upon  =  Avg Diff change factor >2.0, and/or p>0.05 for pairwise comparisons with mock-infected liver tissue.

### Microfluidic card system mRNA assessment

From the overexpressed genes, 18 genes were subsequently investigated with a TaqMan® Custom Array microfluidic card system in order to assess respective expression status at the mRNA level in reference to 5 reference genes (Gapdh, Est1, Rlp3, Mdh-1, Rpl37). In Table 2, up- and downregulation were calculated from real time data, genes were clustered according to the mode used for Table 1.

**Table 2:** TaqMan® Custom Array microfluidic card system was used to assess expression status at the mRNA level of 18 selected genes (see Table 1) in reference to 5 reference genes (Gapdh, Est1, Rlp3, Mdh-1, Rpl37).

#	REFSEQ ID		GENE	*Gapdh* contr./inf.	*Est1* contr./inf.	*Rpl23* contr./inf.	*Mdh-1* contr./inf.	*Rpl-37* contr./inf.
**OVEREXPESSED/UPREGULATED**								
*Immune response/defense* [functional group]: Macrophages (MØ)								
02	NM_011579		Tgtp	9.7	8.2	8.8	11.1	10.7
08	NM_010766		Marco	6.3	5.1	5.6	7.0	6.8
12	NM_020008		Clec7a	4.0	3.3	3.7	4.5	4.4
13	NM_013563		Il2rg	3.7	3.2	3.5	4.2	4.2
14	NM_010705		Lgals3	3.5	3.0	3.3	4.1	3.9
10	NM_009283		Stat1	2.9	2.5	2.7	3.3	3.4
*Immune response/defense* [functional group]: APC								
17	NM_011579		H2-Eb1 (Ab1)	6.8	5.9	6.3	7.8	7.5
19	NM_010386		H2-Dma (H2aa)	5.9	5.0	5.4	6.7	6.4
20	NM_010387		H2-DMb1 (H2-Eb1)	6.3	5.3	5.7	7.1	6.7
21	NM_001042605		Cd74	5.7	4.9	5.3	6.6	6.2
*Immune response/defense* [functional group]: APC								
22	NM_008599		Cxcl9	6.5	5.5	5.6	7.3	7.0
23	NM_207105		Ccl5	6.0	5.1	5.9	7.0	6.8
24	NM_021274		Cxcl10	6.2	5.0	5.0	6.9	6.2
25	NM_021718		Ms4a4b	4.8	3.8	4.2	5.3	5.2
29	NM_008013		Fgl2	3.4	2.8	3.1	3.8	3.7
28	NM_010745		Ly86	3.3	2.6	2.8	3.6	3.4
30	NM_011693		Vcam1	3.0	2.6	2.9	3.5	3.5
*Intermediary metabolism* [functional group]: hepatocytes								
35	NM_008509		Lpl	3.5	2.9	3.5	4.0	4.0
CONTROLS			18s	1.5	1.1	1.2	*1.6*	1.5
standards			Gapdh	***1.0***	1.6	1.7	1.7	1.7
			Est1	2.0	***1.0***	1.8	2.2	2.3
			Rlp23	1.7	1.4	***1.0***	1.9	2.0
			Mdh-1	1.5	1.2	1.3	***1.0***	1.7
			Rpl37	1.5	1.3	1.4	1.7	***1.0***

Values correspond to mean of 5 infected animals (inf.) or 3 non-infected control animals (contr.).

When comparing microarray and microfluidic card system, they exhibited a high level of congruency (Spearman correlation rho = 0.81, p = 7.87e-5).

### Overview of differential gene regulation

Most (34 from 36, 94%) of upregulated genes were annotated to <defense and immune response> processes, while 2 genes (6%) were annotated to <intermediary metabolism> of hepatocytes. Upregulated genes associated with <immune response/defense> (n = 34) could be clustered into functional subgroups including <macrophages> (n = 15), <APCs> (n = 6), <lymphocytes, chemokines and regulation> (n = 10), <B-cells> (n = 2) and <eosinophils> (n = 1). Two downregulated genes related to <lymphocytes, chemokines and regulation> and <intermediary metabolism>, respectively (Tab. 1).

**Figure 1:**
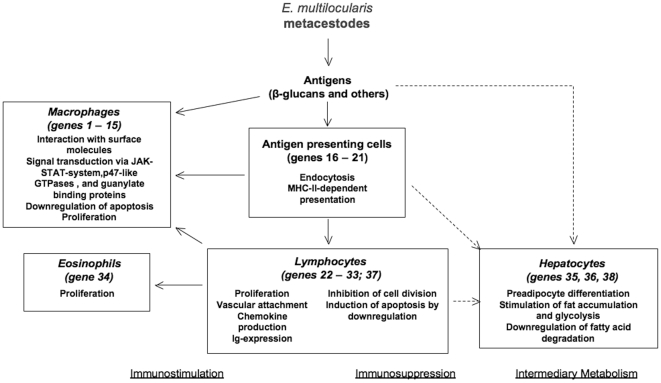
Schematic hepatic cell type interaction profile linked to differentially expressed genes. Interaction of cell types in mouse livers infected with *E. multilocularis* metacestodes as suggested by the pattern of differentially expressed genes (numbers in brackets) shown in Table 1. Interactions indicated by dashed arrows are hypothetical.

The <immune response> genes (n = 35) either associated with an <immunosupression> or an <immunostimulation> pathway, are schematically drawn in (Fig. 1).

In the following paragraph, first upregulated, then downregulated genes will be presented more in details:

### Immune response/defense [MØ]; up-regulated

#### BC023105, Tgtp, Gvin1

These belong to the interferon-inducible p47 GTPases. Interferons (with a preference IFN-γ over α/β) bind to their respective receptors. For Tgtp, the binding is followed by phosphorylation of STAT1 and rapid translocation of P-STAT1 dimers to the nucleus. There, the complex activates transcription of p47 GTPases by binding to the IFN-γ activation site (GAS) in the promoter. The gene products enable the MØ phagosome to be part of the innate defense of cells to infection, but its role in such defense has not yet been clearly defined [6].

#### GBPs (GBP1, GBP2, 5830443L24Rik, GBP8)

GBPs are upregulated in macrophages in response to IFN-γ. GBP-1 is a large GTPase that is induced by inflammatory cytokines and acts antiangiogenically through the inhibition of endothelial cell proliferation and migration [7]. Interestingly, some GBPs are upregulated in mice infected with intracellular pathogens such as *L. monocytogenes* and *T. gondii* and are colocalized with *T. gondii* in infected cells [8]. GBP-1 is also a marker of inflammation that may protect against epithelial apoptosis induced by inflammatory cytokines and subsequent loss of barrier function [9].

#### MARCO

MØ express several host-defense receptors that can be divided into two classes; those dependent on opsonizing components for recognizing pathogens, and those that can recognize pathogens directly. MARCO belongs to a family of class A scavenger with pattern recognition receptors, some of which have been shown to bind lipopolysaccharide that are surface components of many infectious organisms [10]. It will be of upmost interest to search for *E. multilocularis* glycans such as Em492 for their interaction potential with MØ via MARCO.

#### STAT1

STAT1 plays an important role in HA-mediated inflammatory processes [11]. It has been demonstrated that IL-27 controls the development of Th17 and iTreg cells via differential effects on STAT1 [12]. One of the main role of STAT1 is in activating GBP2 transcription to provide transcriptionally competent chromatin [13]. Activation of STAT1 attenuates liver fibrosis through inhibition of HSC proliferation, attenuation of TGF-beta signaling, and stimulation of NK cell killing of activated HSCs. STAT1 could be a new therapeutic target for treating alveolar echinococcosis, similar to that shown e.g. for liver fibrosis [14]. Analysis of T cell responses revealed that STAT1 was not required for the development of a Th1 response, but was required for the infection-induced up-regulation of T-bet. Moreover, Stat1 interacts with Mcm5 and thus may trigger IFN-dependent cell proliferation [15].

#### Mcm5 (CD46)

Mcm5 is a member of the MCM complex which is essential for the initiation of replication. Mcm5 is one of the nuclear proteins that inertact with phosphorylated STAT1 and therefore may play a role in JAK-STAT-modulated cell proliferation [15]. Like other replication factors, Mcm5 is considered as a marker for tumor proliferation, thus it will be interesting to study its putative role in promoting the unrestricted metacestode proliferation encountered in murine alveolar echinococcosis of the liver.

#### Il2rg (CD132), Pleckstrin, Lgals3

Naive T cells can be induced to undergo homeostatic proliferation of variable speed with a few members of the common gamma-chain (CD132) family of cytokines [16]. The IL2 receptor γ chain interacts with insulin receptor substrate (IRS) proteins. These proteins have a pleckstrin binding domain and are phosphorylated partly by JAK-kinases [17]. Pleckstrin is involved in intracellular signaling, e.g. by PI3K pathways that participate in IL-signalling [17], insulin signaling and inflammatory responses [18]. Galectin-3 is a 30 kDa lectin binding β-galactoside expressed and secreted by macrophages. It is a chemoattractant for monocytes and macrophages. CD98 is the receptor for galectin on the macrophage membrane and triggers macrophage activation by the PI3K-pathway. Both components are involved in alternative macrophage activation by IL-4 since disruption of the galectin gene in mice restrains macrophage activation [19].

#### Clec7a

The c-type lectin 7a (Dectin-1) is expressed on monocytes, macrophages, and dendritic cells as a phagocytic receptor for β-glucan containing particles. Dectin-1 collaborates with toll-like receptor 2 in inflammatory responses against microbial pathogens [20].

#### Serpina3g

Serpin3g is a cytoplasmic inhibitor of papain-like, and is required for the protection of cells from caspase-independent PCD triggered by tumor necrosis factor-alpha. In the absence of caspase activity, Spi2A suppressed PCD by inhibiting cathepsin B after it was released into the cytoplasm. Spi2A also directly protects against ROS-mediated PCD, which is consistent with a role in suppressing caspase-independent pathways of PCD. Inhibition of lysosomal executioner proteases by Spi2A is a physiological mechanism by which cells are protected from caspase-independent programmed cell death [21].

### Immune response/defense [APC]; up-regulated

#### H2-Ab1, H2-Eb1, H2-aa, H2-Dma, H2-DMb1

Activation of naive CD4 T cells by dendritic cells requires the sequential interaction of many TCR molecules with peptide-class II complexes of the appropriate specificity. Such interaction results in morphological transformation of class II MHC-containing endosomal compartments. It is generally accepted that MHC II alleles may influence T-cell functions by restricting TCR access to specific residues of the I-A-bound peptide. Thus, this is of significance to diseases that display genetic linkage to specific MHC II alleles, which, however, has not yet been demonstrated for neither murine nor human alveolar echinococcosis.

#### CD74

Dendritic cells (DCs) sample peripheral tissues of the body in search of antigens to present to T cells. This requires two processes, antigen processing and cell motility, originally thought to occur independently. The major histocompatibility complex II-associated invariant chain (Ii or CD74), a known regulator of antigen processing, negatively regulates DC motility in vivo [22].

### Immune response/defense [Lymphocytes, chemokines and regulation]; up-regulated

#### CXCL9, CXCL10

CXCL9 is a proinflammatory monokine, induced by interferon-gamma, which supports Th1-cell mediated tissue inflammation [23]. CXCL10 regulates liver innate immune response [24], its role in alveolar echinococcosis is still unknown. It is generally accepted to potentiate the gene expression of iNOS and CXC chemokine ligand 10 (CXCL10), a major chemoattractant of T helper cell type 1 [25]. This protein is also expressed as a marker of hepatic inflammation and injury, suggesting a role in liver repair and regeneration [26].

#### CCL5 (RANTES)

CCL5 expression correlates with resistance, and blockade of CCL5 rendered mice more susceptible to infection. CCL5 is part of the cascade of events leading to efficient parasite control such as in *L. major* infection. CCL5 up-regulates IL-12, IFN-gamma, and migration of Th1 cells, particularly memory T cells [27].

#### Ms4a4b

MS4a4B was reported to be expressed in Th1-cells but not Th2-cells. Overexpression of MS4a4B in primary CD4+ T-cell blasts enhanced T-cell receptor (TCR)-induced Th1 cytokine production. This suggested that MS4a4B expression is tightly regulated during T-cell development and that MS4a4B expression promotes Th1 function and/or differentiation [28].

#### Cdkn1a (P21)

P21 plays an essential role in determining the type of cell death, positively for apoptosis and negatively for autophagy [29]. Genetic inactivation of p21 in JNK1−/− mice restored hepatocyte proliferation in models of both liver carcinogenesis and liver regeneration, and overexpression of c-Myc increased proliferation of JNK1−/− liver cells. Pharmacologic inhibition of JNK reduced the growth of both xenografted human HCC cells and chemically induced mouse liver cancers. These findings provide a mechanistic link between JNK activity and liver cell proliferation via p21 and c-Myc and suggest JNK targeting can be considered as a new therapeutic approach for HCC treatment. [30].

#### Ifi205

Ifi205 belongs to the class of interferon inducible p200 proteins that regulate cell proliferation. Ifi205 has been shown to upregulate the cell cycle inhibitor P21 by interacting with p53 [31].

#### Ly86 (MD1)

Ly86 contributed to LPS-induced B-cell proliferation, antibody production, and B7.2/CD86 up-regulation [32].

#### FGL2

FGL2 inhibits dendritic cell maturation and induces apoptosis of B cells through binding to the low-affinity FcgammaRIIB receptor, and thus contributes to Treg cell activity [33]. There is evidence FGL2 exerts immunosuppressive effects on T cell proliferation and DC maturation [34].

#### VCAM-1

Vascular cell adhesion molecule-1 (VCAM-1) mediates cell adhesion and transendothelial migration of leukocytes. These molecules do not play a direct role in the recruitment of leukocytes to the infected liver, but instead contribute to IL-12p40-production by splenic CD8(+) dendritic cells (DC). This can be associated with reduced anti-parasitic CD4(+) T cell activation in the spleen and lowered hepatic IFN-gamma, TNF and nitric oxide production. Such effects can be associated with enhanced parasite growth in the liver [35].

#### Rgs2 (29)

Rgs (regulator of G protein signaling) proteins have been characterized as inhibitors of signal transduction cascades initiated by G-protein coupled receptors. Rgs2 is widely expressed in mouse and human tissues. Knock-down studies have shown that Rgs2 is important for T-cell-proliferation and interleukin production [36].

### Immune response/defense [eosinophils]; up-regulated

#### Ear2 (EDN)

Based on its ability to serve as a chemoattractant and activator of DCs, as well as the capacity to enhance antigen-specific immune responses, Ear2 (eosinophil-associated ribonuclease 4) is considered to have the properties of an endogenous alarmin that alerts the adaptive immune system for preferential enhancement of antigen-specific Th2 immune responses [37]. Furthermore, anti-microbial actiovities have been documented by Nakajima et al. [38]. Mouse EAR2, is also chemotactic for human as well as mouse DCs [39].

### Intermediary metabolism [hepatocytes]; up-regulated

#### Lpl

Lipoprotein lipase (Lpl) binds to lipoproteins and specific cell surface proteins in a non-catalytic way. Lpl is expressed in a tissue-specific pattern during development with an increase in adipose tissue, but a decrease in liver. Interestingly, Lpl is also expressed in macrophages where interleukins and interferons downregulate and free fatty acids upregulate expression [40]. Conclusively, an increase would suggest the differentiation of adipocytes in the infected liver.

#### Hk3

Hexokinase III has a very high affinity for glucose, is inhibited by glucose at higher concentrations. Given the specific catalytic patterns, hexokinase III is most likely involved in anabolic processes, e.g. lipid biosynthesis, by providing NADPH by the pentose phosphate pathway [41].

### Immune response/defense [Lymphocytes, chemokines and regulation]; down-regulated

#### Pim3

Pim3 is expressed at low levels in the liver, but upregulated in malignant liver tissue [42]. Pim 3 phosphorylates and thus inactivates the pro-apoptotic protein Bad. The active Bad protein binds to anti-apoptotic proteins of the Bcl2 family thus allowing induction. By phosphorylation of Bad, binding sites for 14-3-3-protein are created. The resulting Bad-14-3-3-complex is no longer able to interfere with Bcl2 proteins thus preventing cell death [43].

### Intermediary metabolism [hepatocytes]; down-regulated

#### Crotonase homologous

Crotonase (Enoyl-coenzyme-A-hydratase) catalyses the hydratation of trans-2-enoyl-CoA thioesters resulting from the first step of beta-oxidation of fatty acids [44].

## Discussion

### Functional analysis, immunostimulatory pathway

The involvement of cellular immunity in controlling the infection is strongly suggested by the intense granulomatous infiltration observed in the periparasitic area of lesions in experimentally infected mice [45], [46]. Immunodeficient athymic nude [47] and SCID mice [48] as well as HIV-co-infected patients [49], [50] exhibited high susceptibility to infection and disease, thus suggesting that the host cell mediated immune response plays an important role in suppressing the larval growth. *E. multilocularis* appears to induce skewed Th2-responses [46]. Based on in vitro and in vivo studies, Th2-dominated immunity was associated with increased susceptibility to disease, while Th1 cell activation through IL-12 [46], IFNγ [51], [52], TNFα [53] and IFNα [54] was suggested to induce protective immunity in AE [55], [56]. Innate mechanisms appeared also to resistance upon attack by cytotoxic compounds such as activated complement proteins and NO, associated to increased macrophage activities [57], [58]. With regard to the hepatic gene expression profiles of our study related to immunostimulatory pathways (thus potentially also to efficient host control mechanisms), most of the highest expressed molecules (BC023105, Tgtp, Gvin1, GBPs, Clec7a) appear to localize within cytokine pathways involved in direct or indirect MØ activities. Respective parasite cell damage may be one option, but also subsequent antigen processing (MARCO: carbohydrates? Ly86: LPS-like antigens?) and orchestration of the subsequent T cell orientation (STAT1, Mcm5, Il2rg, Pleckstrin, Lgals3). Beside MØ, dendritic cells may intrahepatically contribute to the immunological response and defense, as indicated by hyperexpressed partner cell (e.g. CD4 T cells) molecules such as H2-Ab1, H2-Eb1, H2-aa, H2-Dma, H2-DMb1, CD74 and CCL5, all associated to CD4 T cell activation and action, especially Th1 (CCL5, Ms4a4b, Rgs2). That all these events are accompanied by periparasitic inflammatory processes are supported by overexpressed CXCL9, CXCL10. Finally, another hyperexpressed molecule (Serpina3g) may synergistically contribute to parasitocidal host effector mechanisms by controlling immunopathological events related to apoptotic tissue damage, putatively triggered by Cdkn1a, and downregulated Pim3 may contribute to such a phenomenon as well.

To carry out a more profound interpretation of the present findings, we have to take into account that the time point of investigation (infection status) corresponds to a rather early stage, not yet switched into the late/chronic stage of AE. Globally, the above listed phenomena correspond mostly to a still Th1-oriented immune response, which may putatively be the correct way to control infection. We know, however, that the parasite survives in the host. By inducing functional changes in DCs and MØs, the metacestode can achieve important shifts in T-cell subsets. The initial acute inflammatory Th1 response is subverted gradually to a Th2 response during the chronic phase of AE [59]. Cytokines, such as IL-4, IL-5, IL-9 and IL-13, secreted largely by immune-cell types in response to parasite antigens, not only down-modulate the Th1 response but can also promote parasite expulsion and tissue renewal and repair [60]. The metacestode most likely achieves the late infection stage Th2 expansion through the induction of regulatory cytokines, such as IL-10 and TGF-β [59]. To provide a platform of understanding the late stage event, we will have to prompt a more detailed investigation by using late stage infection mice. The question will arise as to whether it will be possible to obtain appropriate hepatic tissue free of metacestode material (a prerequisite in the present study), as at the advanced/late stage, usually the whole liver is metastatically interspersed with parasite cells/vesicles. One of the key questions remaining is how during the infection course the initial Th1-orientation switches to a rather Th2-oriented pathway. Our interest will thus focus on unraveling the metacestode tools (metabolites) that could trigger such a re-orientation.

### Functional analysis, immunosuppressive pathway

An interesting feature observed in chronic AE is a marked depression of the cell-mediated immune response [61], [62], [63]. These general characteristics of *E. multilocularis*, including the seemingly tumor like growth, its ability to modulate host immune responses, and the fact that in vitro culture is an established technique, renders this parasite a very attractive model to study the host-parasite interplay in view to reveal potentially novel modes of therapy [56]. Increased concentrations of the pro-inflammatory cytokines TNF-a, IL-1b, iNOS and the anti-inflammatory IL-I0 are characteristic for secondary AE in mice [58].

With regard to the hepatic gene expression profiles of our study related to immunosuppressive pathways (thus potentially also to the support of parasite survival capacities), overexpressed molecules such as FGL2 can already interfere at early innate stage by inhibiting dendritic cell maturation, or by reducing T cell activation (VCAM-1). Such effects can be associated with enhanced parasite growth in the liver [64]. Interestingly, like in many other helminthic infections, the host immune response seems to make use of a rather Th2-dependent eosinophilic component to trigger its fight against the infectious agent (Ear2, EDN). The mobilization of eosinophils is known to be a crucial immunological event that plays an important role in the host defense against helminths. Despite appropriate host signals, there is a marked lack of eosinophilic infiltrates in experimental murine AE [59]. One possible explanation was recently provided by the demonstration that *E. multilocularis* metabolites exhibit proteolytic activity on eotaxin in vitro [58]. Inhibition of eotaxin activity may suppress the mobilization of eosinophils in *E. multilocularis*-infected mice. Absent eosinophils thus may be a part of a series of events that maintain a low level of inflammation in *E. multilocularis*-infected hosts.

### Hepato-pathogenesis

The pathogenesis of liver damage in AE arises from the interplay of the parasitic metacestode and the host inflammatory response. It is generally accepted that the release of parasitic products/metabolites (such as cysteine proteases [59]) into the periparasitic hepatic tissue triggers the inflammatory response in the liver parenchyma by inducing the production and release of inflammatory cytokines, chemokines and lipid inflammatory mediators [59]. At an early stage of infection, little liver damage is observed [65]. That may be the reason why our study yielded few deregulated genes related to hepatocyte functions. The upregulation of a lipoprotein lipase and a hexokinase most likely involved in anabolism and the downregulation of crotonase are indications for a metabolic reprogramming of hepatocytes (Fig. 1) in the direction of fat accumulation (differentiation of adipocytes). Recently, an apolipoprotein binding protein from *E. multilocularis* hydatid fluid has been characterized [66]. These findings indicate the parasite may act as a sink for host lipids and stimulates lipid accumulation in vicinal host tissue. It is, however, unclear whether *E. multilocularis* is able to use lipids as a source for carbon and energy. Although it is clear that glycolysis and tricarboxylic acid cycle are functional (yielding succinate under anaerobic conditions), results concerning lipid catabolism via beta-oxidation are lacking [67]. Host lipids may also be attracted and exposed as a part of a strategy to circumvent the host immune system. In any case, these proteins could be interesting targets to address in future studies, presently these molecules remain still at a speculative level and are difficult to discuss without further pathological characteristics related to these molecules. At a late stage of infection, fibrosis becomes a hallmark of AE, leading to a complete disappearance of the liver parenchyma and even to the death of the metacestode, with vesicles embedded in an acellular tissue composed nearly entirely of cross-linked collagens [68]. The diffusion of the fibrotic process even far from the parasitic lesions strongly suggests a major role for cytokines in collagen synthesis. But these features could not be addressed and thus also not discussed in the present study. As mentioned above, we will design new studies respecting temporal aspects to unravel late stage characteristics of murine AE.

### Conclusion

The conventional course of AE as a disease in humans resembles strongly that of the naturally infected mouse, in that untreated AE will, in many but not most cases, finally lead to fatality.

In order to better understand periparasitic events characterizing the host response to infection, we assessed the gene expression profile in the periparasitic liver tissue during early chronic AE. High throughput analysis of gene expression yielded a set of mostly immunologically related upregulated genes, while downregulation almost exclusively lacked. The data presented herein may provide a road map for further investigations into the pathophysiology of AE and may help to identify potential targets for adjuvant therapy of this disease. Future interest will also focus on unraveling similar events but at late stage infection.

## Methods

### Model of alveolar echinococcosis

An established mouse model of primary alveolar echinococcosis was used as previously described [3], [69]. The animal studies were approved by the Animal Care and Experimentation Committee of the Canton of Bern, Switzerland, and followed National Institutes of Health guidelines for the performance of animal experiments. Briefly, 8-weeks-old female C57BL6/J mice were purchased from Charles River GmbH (Germany), and infected (n = 10) at the age of 10 weeks by peroral inoculation with 100 µl sterile water containing 2×10^3^ eggs of *E. multilocularis*, using appropriate biosafety level 3 laboratory conditions (Swiss biosafety approval number A990006/3A). The infecting organisms (parasite eggs) were initially isolated from a naturally infected fox. Egg viability and infectiosity were pre-evaluated upon explorative titrated infection experiments carried out in mice preliminarily to the present studies [4]. Mock-infected control animals (n = 5) were perorally inoculated with 100 µl of sterile water. 31 days after infection, the animals were weighed and the infection intensity of AE was scored by counting the number of parasitic lesions macroscopically visible on and within the liver tissue [4]. Animals were sacrificed with an overdose of pentobarbital (100 mg/kg, intraperitoneally) for the mock-infected control group (n = 5) and for the group representing the chronic stage of primary AE (n = 10).

### Tissue processing

Animals were perfused via the left cardiac ventricle with 30 ml of ice-cold, RNase-free phosphate buffered saline (PBS) followed by 30 ml of 50% RNAlater@ (Ambion Europe Ltd., Huntingdon, UK) in ice-cold, RNase-free PBS. Immediately afterwards, the liver was removed. Approximately 5 mm^3^–sized periparasitic liver tissue blocks (adjacent by 1 mm to the macroscopically visible parasitc lesion) were dissected and stored separately in 150 µl of RNAlater® at 4°C until isolation of RNA.

### RNA processing and microarray hybridization

Liver tissue samples of each animal were processed and analyzed separately. Total RNA was extracted from liver tissue using RNeasy® Lipid Tissue kit (QIAGEN, Basel, Switzerland) and purified with RNeasy columns (QIAGEN, Basel, Switzerland). Quantification and assessment of RNA integrity were performed on the Agilent 2100 bioanalyzer platform (RNA 6000 Nano, Agilent technologies, Waldbronn, GER) and validated on the NanoDrop® (NanoDrop, Wilmington, USA) quantification device. Based on RNA quality control results and histopathological evaluation of hippocampal damage (apoptosis score) RNA extracts from 3 infected and 3 control animals were selected for each time point for array hybridization. Double-stranded cDNAs were synthesized from 5 µg of total RNA using an oligo dT-T7 promoter primer (Roche Molecular Biochemicals, Mannheim, Germany). The cDNAs obtained were used as templates for in vitro transcription using the Megascript kit purchased from Ambion (Austin, TX) and biotinylated nucleotides (Bio-11-CTP and Bio-16-UTP) provided by Roche Molecular Biochemicals (Basel, Switzerland). Fragmented in vitro transcripts (cRNAs) were hybridized overnight on to commercially available rat microarrays (GeneChip® Rat Genome 230 2.0 Array, Affymetrix, Santa Clara, CA) containing 31'000 probe sets representing approximately 28'000 well-substantiated rat genes. The hybridized samples were stained with streptavidin-R phycoerythrin (SAPE, Molecular Probes Inc., Eugene, OR) and the signal was amplified using a biotinylated goat anti-streptavidin antibody (Vector Laboratories, Burlingame, CA). Washing, staining and amplification were carried out in an Affymetrix GeneChip® Fluidics Station 450. Microarrays were scanned in an Affymetrix GeneChip® scanner 3000. Signal intensities were calculated based on image files with the Affymetrix GeneChip® Operating software (GCOS) v1.1.1.

### Raw data analysis

Chip data analysis was performed on the R platform for statistical programming using packages of the Bioconductor project [70]. Due to the asymmetric distribution of microarray data all datasets were log2 transformed. Background correction, normalization and data summary were performed with non linear methods using the rma function of the affy package [71]. Chip quality assessment was exploratively performed based on boxplots of the raw log scale intensities and MA-plots visualizing signal intensity dependent effects on the log-ratios. Additionally the quality of the hybridized cRNA was assessed by RNA digestion plots where the mean intensities of all probes on an array are plotted according to their 5′ to 3′probeset position (affy package).

To reduce the number of hypothesis to be tested in the subsequent significant tests, genefiltering was perfomed. All genes that were expressed under the estimated background intensity of 60 fluorescent units (FU) on at least 4 of the 8 chips and genes showing an interquantile range (IRQ) of less than 0.5 were excluded from significance testing.

Significance testing was performed using the limma package [72]. Type 1 error was corrected by the Benjamini-Hochberg false discovery rate algorithm [73].

All raw data is MIAME compliant and have been deposited in “ArrayExpress” (EMBL/EBI) (accession no. E-MEXP-2615).

### Custom-designed TaqMan® Gene Expression Assays

In order to obtain an *ex vivo* confirmation of the Affymetrix gene expression findings, we selected 18 (upregulated) out of the 36 genes initially differentially expressed, to subject them to a custom-designed real-time PCR Applied Biosystems 7900HT Micro Fluidic Card system. For this, TaqMan Low Density Arrays using 384-well micro fluidic cards were pre-loaded with a selection (Table 3) of differentially expressed genes (selection was primarily based upon the availability of standardized primer pairs to quantitatively investigate respective gene expression).

**Table 3:** Selected list of genes and respective primer sets to assess expression status at the mRNA level using a TaqMan® Custom Array microfluidic card system.

#	gene description	Applied Biosystems primer sets
01	T-cell specific GTPase	Tgtp-Mm00786926_s1
03	histocompatibility 2, class II antigen A, beta 1	H2-Ab1-Mm00439216_m1
04	histocompatibility 2, class II antigen E beta	H2-Eb1-Mm00439221_m1
07	histocompatibility 2, class II antigen A, alpha	H2-Aa-Mm00439211_m1
08	chemokine (C-X-C motif) ligand 9	Cxcl9-Mm00434946_m1
09	chemokine (C-C motif) ligand 5	Ccl5-Mm01302427_m1
10	chemokine (C-X-C motif) ligand 10	Cxcl10-Mm00445235_m1
13	macrophage receptor with collagenous structure	Marco-Mm00440265_m1
16	CD74 antigen (invariant polypeptide of MHC class II)	Cd74-Mm00658576_m1
19	lymphocyte antigen 86	Ly86-Mm00440240_m1
20	vascular cell adhesion molecule 1	Vcam1-Mm00449197_m1
21	membrane-spanning 4-domains, subfamily A, member 4B	Ms4a4b-Mm00649916_m1
24	fibrinogen-like protein 2	Fgl2-Mm00433327_m1
25	signal transducer and activator of transcription 1	Stat1-Mm00439518_m1
30	lipoprotein lipase	Lpl-Mm00434764_m1
31	lectin, galactose binding, soluble 3	Lgals3-Mm00802901_m1
36	C-type lectin domain family 7, member a	Clec7a-Mm00490960_m1
38	RIKEN cDNA 1810022C23 gene	18S-Hs99999901_s1

Relative quantification was based on the reference of the following 5 constitutively expressed reference genes (Table 4). These genes were selected from the microarray data based on i) their minimal variability between the control and infection group ii) their absolute expression level covering the whole array signal range.

**Table 4:** The custom-designed TaqMan® Gene Expression Assays were quantified upon use of 5 constitutively expressed reference genes and corresponding primer sets, respectively.

gene description	gene	Applied Biosystems primer sets
Glyceraldehyde-3-Phosphate Dehydrogenase	GAPDH	Gapdh-Mm99999915_g1
Mitochondrial resident protein ES1	ES-1	Es1-Mm00468347_m1
Ribosomal protein 23	RPL-23	Rpl23-Mm00787512_s1
Malate dehydrogenase 1	MDH1	Mdh1-Mm00485106_m1
Ribosomal protein 37	RPL-37	Rpl37-Mm00782745_s1
